# Body Condition and Temperature–Humidity Index Are Associated With Cortisol Levels as Indicators of Physiological Stress in Sumatran Elephants

**DOI:** 10.1155/vmi/1184315

**Published:** 2026-06-08

**Authors:** Arman Sayuti, Yudha Fahrimal, Amalia Sutriana, Cristopher R. Stremme, Riyan Ferdian, Ridwan Ridwan, Taufiq Purna Nugraha, Gono Semiadi, Gholib Gholib

**Affiliations:** ^1^ Graduate School of Mathematics and Applied Sciences, Universitas Syiah Kuala, Banda Aceh, 23111, Aceh, Indonesia, unsyiah.ac.id; ^2^ Laboratory of Clinic, Faculty of Veterinary Medicine, Universitas Syiah Kuala, Banda Aceh, 23111, Aceh, Indonesia, unsyiah.ac.id; ^3^ Center for Wildlife Study, Faculty of Veterinary Medicine, Universitas Syiah Kuala, Banda Aceh, 23111, Aceh, Indonesia, unsyiah.ac.id; ^4^ Laboratory of Parasitology, Faculty of Veterinary Medicine, Universitas Syiah Kuala, Banda Aceh, 23111, Aceh, Indonesia, unsyiah.ac.id; ^5^ Laboratory of Pharmacology, Faculty of Veterinary Medicine, Universitas Syiah Kuala, Banda Aceh, 23111, Aceh, Indonesia, unsyiah.ac.id; ^6^ Aceh Conservation and Resources Agency, Ministry of Environment and Forestry, Banda Aceh, Aceh, Indonesia; ^7^ Research Centre for Applied Zoology, National Research and Innovation Agency, Bogor, Indonesia, brin.go.id; ^8^ Research Centre for Biosystematics and Evolution, National Research and Innovation Agency, Bogor, Indonesia, brin.go.id; ^9^ Physiology Laboratory, Faculty of Veterinary Medicine, Universitas Syiah Kuala, Banda Aceh, 23111, Aceh, Indonesia, unsyiah.ac.id

**Keywords:** body condition score, cortisol, physiological stress, Sumatran elephant, temperature–humidity index

## Abstract

The Sumatran elephant (*Elephas maximus sumatranus*), a critically endangered subspecies endemic to Indonesia, is increasingly exposed to physiological stress associated with habitat disturbance and environmental conditions. Reliable assessment of stress physiology is therefore essential to support evidence‐based welfare monitoring and conservation management, particularly for elephants maintained in conservation rescue units (CRUs). This study aimed to (1) validate a cortisol enzyme‐linked immunosorbent assay (ELISA) kit for use in Sumatran elephants and (2) apply the validated assay to examine the effects of body condition score (BCS), ambient temperature, humidity, and temperature–humidity index (THI) on cortisol levels across CRUs in Aceh Province, Indonesia. Twenty elephants (8 males and 12 females; 10–49 years old) housed at six CRUs (CRU A–F) were included. Analytical validation assessed parallelism, accuracy, and assay precision, while biological validation compared cortisol levels during medical treatment and postrecovery. Factors influencing cortisol levels were evaluated using linear mixed‐effects models incorporating BCS, location, ambient temperature, humidity, and THI as fixed effects, with elephant identity as random effects. The assay demonstrated acceptable analytical performance, with strong parallelism between serially diluted samples and the cortisol standard curve. Biological validation showed significantly higher cortisol levels during medical treatment than during recovery (*p* < 0.01). Mixed‐effects analyses showed that BCS, location, ambient temperature, humidity, and THI were significantly associated with cortisol levels (*p* < 0.01). Elephants with BCS 7 (scale 1–9) tended to exhibit lower cortisol levels. Variation in cortisol levels was also observed among locations. Cortisol levels tended to increase with higher ambient temperature and THI, whereas higher humidity was associated with lower cortisol levels. In conclusion, the validated cortisol assay demonstrated suitability for use in Sumatran elephants under the conditions of this study. The findings suggest that body condition and environmental factors (e.g., ambient temperature, humidity, and THI) may be associated with variation in cortisol levels in elephants managed in CRUs, suggesting the importance of considering both physiological and environmental contexts in welfare monitoring.

## 1. Introduction

The Sumatran elephant (*Elephas maximus sumatranus*) is a subspecies of the Asian elephant (*Elephas maximus*) and is endemic to Sumatra Island, Indonesia. Over the past few decades, habitat loss, poaching, and increasing human–elephant conflict have led to a significant decline in population and extensive fragmentation of remaining habitats. Consequently, the Sumatran elephant has been classified as Critically Endangered by the International Union for Conservation of Nature (IUCN) since 2011 [1]. Current estimates indicate that approximately 1724 individuals are left in the wild, and around 243 individuals are in managed care in Indonesia [2]. Alarmingly, population monitoring data show a reduction of nearly 700 individuals between 2011 and 2017, highlighting the urgent need for effective conservation and management strategies.

Conservation rescue units (CRUs), which serve as semimanaged facilities intended to reduce immediate threats like habitat encroachment, poaching, and human–elephant conflict, are home to a significant number of Sumatran elephants in Aceh Province. Elephants in these facilities receive routine veterinary care and health monitoring, including regular antihelmintic treatment, contributing to improved physical condition and overall welfare. However, despite these interventions, elephants under managed care may still experience physiological stress associated with environmental factors, management practices, and nutritional status [3]. Therefore, assessing stress and its determinants remains a critical component of conservation physiology for this species.

Body condition score (BCS) is widely used as an indicator of an animal’s nutritional status, energy reserves, and overall health and may be associated with physiological responses to stressors [4]. Individuals with low body condition may exhibit reduced physiological resilience, rendering them more vulnerable to stress‐induced dysregulation of the hypothalamic–pituitary–adrenal (HPA) axis [5]. Prolonged or excessive activation of the HPA axis can result in elevated cortisol secretion, which may have deleterious effects on immune function, metabolism, and reproductive performance [6]. Conversely, while animals with higher body condition may have greater energy reserves, excessive body condition has also been associated with adverse physiological outcomes, including metabolic imbalance, impaired reproductive performance, and altered endocrine function [7, 8]. Therefore, both low and excessively high body condition may influence stress physiology, highlighting the importance of maintaining an optimal body condition range.

Environmental conditions, particularly ambient temperature and humidity, may influence physiological stress responses in large mammals [9]. Elephants are highly sensitive to thermal stress due to their large body mass and limited capacity for evaporative cooling [10]. However, they exhibit a range of thermoregulatory mechanisms to cope with heat stress, including behavioral and physiological strategies such as ear flapping, mud bathing, and vascular heat dissipation through highly perfused ear tissues, which facilitate heat loss and help maintain body temperature [10, 11]. Despite these adaptations, elevated ambient temperatures and humidity may still increase thermoregulatory demands and metabolic load, which can influence endocrine responses, including cortisol secretion [12]. In addition to individual environmental variables, THI is widely used as an integrative indicator of thermal stress, reflecting the combined effects of temperature and humidity on heat load. In this context, body condition may further contribute to variability in these responses. Understanding the combined influence of body condition and thermal environment is therefore important for interpreting stress physiology in elephants under managed and seminatural conditions.

Physiological stress in elephants is commonly assessed through measurement of cortisol concentrations, a key glucocorticoid hormone released following HPA axis activation [13]. Enzyme‐linked immunosorbent assay (ELISA) techniques are widely used for cortisol quantification. However, any assay used must be rigorously validated for each target species and biological matrix to ensure accuracy and biological relevance [14, 15]. Validation typically involves both analytical validation (assessing assay sensitivity, precision, and accuracy) and biological validation, which confirms that measured hormone concentrations reflect biologically meaningful physiological changes [16].

Although cortisol assays have been validated for Asian elephants [3, 4, 13], information on their validation in Sumatran elephants, particularly under managed care conditions, remains limited. In addition, differences in assay kits, sample matrices, and laboratory conditions may influence assay performance; therefore, context‐specific validation before application is warranted. The objectives of the present study were to (1) validate a cortisol ELISA kit for use in Sumatran elephants and (2) apply the validated assay to evaluate the associations between body condition, ambient temperature, humidity, and THI with cortisol levels in elephants housed across CRUs in Aceh Province, Indonesia. It was hypothesized that the assay would be suitable for this species and that cortisol levels would be associated with BCS and environmental variables, including temperature, humidity, and THI.

## 2. Materials and Methods

### 2.1. Study Site and Animals

This study was conducted at CRUs, managed care facilities for Sumatran elephants in Aceh Province, Indonesia. A total of 20 captive Sumatran elephants, comprising 8 males and 12 females, were included in the study. The elephants ranged in age from 10 to 49 years during the study period (Table 1). The animals were housed across six CRUs (CRU A–F) distributed across diverse environmental settings that differed in habitat type and elevation. CRU A (44 m above sea level) was located in a mixed forest and oil palm (*Elaeis guineensis*) plantation landscape. CRU B (75 m above sea level) was located in a fragmented lowland habitat. CRU C (257 m above sea level) was positioned within a highland forest–coffee agroecosystem, whereas CRU D (21 m above sea level) was situated within the Ulu Masen forest at low to moderate elevation. CRU E (14 m above sea level) was located within the Leuser forest landscape adjacent to plantation areas, and CRU F (395 m above sea level) represented a seminatural managed environment at a higher elevation. Overall, the study sites encompassed a gradient of lowland to upland ecosystems, including forest, agroforestry, and semimanaged habitats.

**TABLE 1:** Characteristics of the Sumatran elephant population studied at conservation rescue units in Aceh, Indonesia.

Conservation rescue units	ID of elephants	Sex	Age range during study period (years)	Range of body condition score	Number of samples
A	E1	Male	21‐22	6‐7	6
	E2	Female	37	7	4
	E3	Female	39‐40	5–7	8

B	E4	Male	29	6‐7	4
	E5	Female	32	6‐7	4

C	E6	Female	18	6‐7	4
	E7	Female	29‐30	5–7	6
	E8	Male	48‐49	7	6
	E9	Female	33‐34	4–7	6

D	E10	Male	30	6‐7	4
	E11	Female	42‐43	6‐7	6
	E12	Female	25	6	4

E	E13	Male	30‐31	5–7	6
	E14	Female	36‐37	4–7	8
	E15	Female	41	4–6	4
	E16	Male	36‐37	4–7	7

F	E17	Male	10‐11	6‐7	6
	E18	Female	24‐25	6‐7	6
	E19	Female	37‐38	5–7	7
	E20	Male	32	7	4

Elephants were maintained under semicaptive management conditions. Each CRU was equipped with a basecamp, and each elephant was assigned to a dedicated mahout responsible for daily care and movement. Elephants were routinely guided to forage within or around the CRU areas and were not grazed in community‐owned plantation lands. During resting and grazing periods, elephants were tethered using chains of approximately 20 m in length, allowing restricted movement while maintaining access to food, water, and shade. Natural water sources, including rivers and ponds, were available at several CRUs and were regularly used for drinking and bathing. In addition to natural foraging, elephants received supplementary feeding consisting of fresh forage and concentrate mixtures formulated from locally available feed resources. Daily management involved regular interactions with mahouts, including feeding, bathing, and routine health monitoring. Direct human interaction beyond standard management practices was limited and controlled, with all activities involving elephants conducted under the supervision of trained mahouts.

Routine health monitoring was conducted quarterly by the Center for Wildlife Study, Faculty of Veterinary Medicine, Universitas Syiah Kuala, in collaboration with the Aceh Natural Resources Conservation Agency. As part of this monitoring program, blood samples and BCS were collected every 3 months over 1‐2 years. A total of 110 blood samples were collected from 20 elephants, with each individual contributing repeated observations (approximately 4–8 samples per elephant), depending on the presence of the elephants at the study sites and the availability of blood sampling (Table 1). No samples were collected from male elephants during musth due to safety considerations and handling constraints; therefore, only nonmusth samples were included in the analysis.

As part of standard veterinary care, all elephants received regular anthelmintic treatment. Deworming was administered quarterly, with dosages adjusted according to individual body weight, using albendazole (2500 mg), closantel (2000 mg), rintal bolus (600 mg), and ivermectin (10 mg).

### 2.2. BCS

**FIGURE 1:**
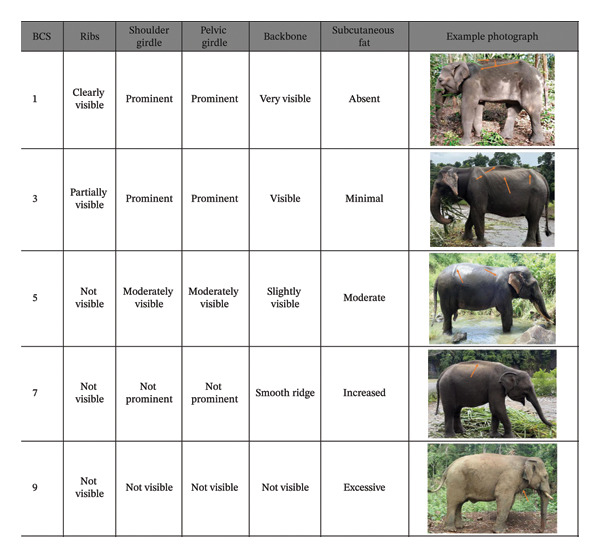
Body condition scoring (BCS) criteria for Sumatran elephants based on anatomical features and subcutaneous fat distribution.

BCS was assessed using a visual scoring system adapted from a previous study in Asian elephants [17, 18], which evaluates external body fat deposition and musculoskeletal prominence based on standardized anatomical landmarks. The scoring system uses a nine‐point scale, ranging from 1 (*emaciated*) to 9 (*obese*), with intermediate scores (3, 5, and 7) representing gradations of body condition. BCS assessments were conducted through lateral visual observation to allow clear visualization of key anatomical regions, including the ribs, shoulder girdle, pelvic girdle, backbone, and subcutaneous fat deposition. All assessments followed standardized criteria and were performed under consistent observation conditions. The scoring criteria and associated anatomical features are summarized in Figure 1.

When an elephant’s condition fell between two defined categories, an intermediate even‐numbered score was assigned. All BCS evaluations were performed by trained mahouts at each CRU who had been previously trained by a veterinarian using a standardized scoring protocol. To ensure consistency and reliability, each assessment was conducted under the supervision of the same veterinarian.

### 2.3. Temperature and Humidity Data Collection

Ambient temperature and relative humidity were measured directly at each CRU at the time of biological sample collection. Ambient temperature was recorded using a calibrated digital thermometer, while relative humidity was measured using a digital hygrometer. For each sampling day, the mean daily ambient temperature and relative humidity were calculated and used as the environmental variables in subsequent analyses. To assess heat stress conditions, the temperature–humidity index (THI) was calculated using the following formula:

(1) THI=1.832×T+−0.550.0055−×RH×1.826×T−,

where *T* is ambient temperature (°C) and RH is relative humidity (%). This formula was adopted from previous studies [19, 20].

### 2.4. Blood Sample Collection

Blood samples were collected from each elephant as part of routine veterinary health examinations. Blood samples were collected by a veterinarian in accordance with established animal welfare protocols to minimize handling stress and discomfort. Blood samples at each CRU were collected in the morning between 08:00 and 10:00 a.m. to control for circadian variation in cortisol levels. Approximately 5 mL of blood was collected from the auricular vein using disposable syringes (5–10 mL) fitted with 18–21‐gauge needles (Terumo, Indonesia) and transferred into appropriate vacutainer tubes for serum separation (Vaculab Plain 5 mL, OneMed, Indonesia). Following collection, blood samples were allowed to clot at ambient temperature and were subsequently centrifuged at 3000 rpm for 10 min to obtain serum. Serum samples were immediately transferred to polypropylene tubes (Eppendorf Safe‐Lock tubes) and stored at −20°C until cortisol analysis. In total, 110 blood samples were collected during the study (approximately 4–8 samples per individual), with sampling performed every 3 months alongside routine health monitoring. Due to logistical considerations, sampling at different CRUs was conducted on different days, and the collection of samples from all sites required approximately 1‐2 weeks (one to two days per CRU). Serum samples were then analyzed within 3 months of storage.

### 2.5. Assay Validation

Before application in this study, the cortisol ELISA kit (Cat No. EIA‐1887, DRG Instruments GmbH, Germany) was validated for use in Sumatran elephants, incorporating both analytical and biological validation procedures [14]. Analytical validation was conducted to evaluate assay performance in terms of parallelism, dose–response characteristics, accuracy, and reproducibility. Parallelism was assessed using serial dilutions (1:2–1:16) of two elephant serum samples (one male and one female), which were assayed concurrently with the cortisol standards. The parallelism test was performed to evaluate the capability of the cortisol ELISA kit to accurately quantify cortisol in the elephant serum matrix by assessing potential matrix interference with antigen–antibody binding. Serial dilutions were used to determine whether substances present in the serum interfered with the interaction between cortisol and the assay antibodies. Parallel displacement between the dilution curves and the standard curve was examined by comparing the slopes of expected dose versus percent bound (%B/B_0_).

Assay accuracy was evaluated using spike‐and‐recovery tests, in which known amounts of cortisol standard were added to elephant serum samples. Recovery percentages were calculated to assess the assay’s ability to accurately quantify cortisol within the biological matrix. Reproducibility was determined by repeated measurement of low‐ and high‐concentration quality controls, both within a single assay run to calculate intra‐assay coefficients of variation (CVs) and across multiple assay runs to determine interassay CVs. Cross‐reactivity and assay sensitivity were defined according to the manufacturer’s specifications. The antibody exhibited 100% cross‐reactivity with cortisol, 45% with corticosterone, 9% with progesterone, < 2% with deoxycortisol and dexamethasone, 0.9% with cortisone, and < 0.01 with estrone, estriol, and testosterone (DRG Instruments GmbH, Germany).

Biological validation was performed using samples from seven elephants (four females and three males) undergoing clinical treatment for injury. These samples were used specifically to evaluate the ability of the cortisol assay to detect physiologically relevant changes in cortisol levels in relation to health status. For each individual, one serum sample was collected during the active treatment phase (injury condition) and one after clinical recovery, resulting in a total of seven samples per condition. Physiologically, cortisol levels are expected to be higher during injury or stress and lower after recovery. This comparison was used to confirm that the assay accurately reflects these biological changes. These samples were used only for biological validation and were not included in analyses of associations with BCS, ambient temperature, or location.

### 2.6. Cortisol Level Measurement

Cortisol levels were measured using a cortisol ELISA kit (Cat. No. EIA‐1887, DRG Instruments GmbH, Germany). All reagents, including wash solution, enzyme conjugate, substrate, and stop solution, were provided within the kit. A volume of 20 μL of standards, quality controls, and undiluted serum samples was dispensed into designated wells of the precoated 96‐well microplate supplied with the kit using fresh disposable pipette tips. Samples were analyzed in duplicate. Subsequently, 200 μL of enzyme conjugate (horseradish peroxidase) was added to each well, and the plate was gently mixed for approximately 10 s to ensure uniform distribution of reagents. The plate was incubated for 60 min at room temperature to allow antigen–antibody binding. Following incubation, the reaction mixture was discarded, and the wells were washed five times with 300 μL of diluted wash solution per well. After the final wash, the plate was inverted and firmly tapped onto absorbent paper to remove any residual liquid. Thereafter, 100 μL of tetramethylbenzidine (TMB) substrate solution was added to each well, and the plate was incubated for 20 min at room temperature in the dark. The enzymatic reaction was terminated by the addition of 100 μL of stop solution (0.5 M H_2_SO_4_) to each well. Absorbance was measured at 450 nm using a microplate ELISA reader (BioLegend, USA). Cortisol concentrations were determined automatically from the standard curve generated by the microplate reader software.

Assay performance showed intra‐assay CVs of 9.52% and 5.14% for low‐ and high‐quality controls, respectively, and interassay CVs of 10.13% and 9.25%. Assay sensitivity was 1.3 ng/mL, as reported in the manufacturer’s protocol.

### 2.7. Data Analysis

All statistical analyses were preceded by assessment of data distribution and variance. Normality and homogeneity were evaluated using the Shapiro–Wilk and Levene’s tests, respectively, and all variables met the assumptions for parametric analyses (*p* > 0.05). For analytical validation, parallelism between serial dilutions of two pooled elephant serum samples and the standard curve was evaluated by comparing the slopes obtained from logarithmic regression analysis using a test of equality. For biological validation, cortisol concentrations during injury treatment and after recovery were compared using a paired‐sample *t*‐test.

To evaluate factors influencing cortisol levels, linear mixed‐effects models (LMMs) were used to account for repeated measures in the dataset. Because each elephant was sampled multiple times (approximately 4–8 observations per individual) over a 1‐ to 2‐year monitoring period, observations were not independent. Moreover, elephant ID was included as a random effect to account for within‐individual correlation and variability in baseline cortisol levels. In the first analysis, BCS and CRU location were included as fixed effects, while elephant ID was incorporated as a random effect to account for repeated measurements within individuals. This analysis was designed to evaluate the association between body condition and cortisol levels while accounting for differences among sites. Because a significant interaction between BCS and location was detected, LMMs were constructed to further investigate the underlying drivers. For further analyses, ambient temperature, humidity, and THI were included as fixed effects, and elephant ID was incorporated as a random effect and was run in separate analyses.

## 3. Results

### 3.1. Assay Validation

Serial dilutions of serum from two elephants generated displacement curves that were parallel to the cortisol standard curve (Figure 2(a)). As anticipated, cortisol concentrations decreased consistently with increasing dilution. Dose–response analysis revealed a slope of −23.242 for the cortisol standard curve, whereas slopes of −21.304 and −21.012 were obtained for serum samples 1 and 2, respectively. Tests for equality of slopes indicated no significant differences among the curves (*p* > 0.05), confirming assay parallelism and demonstrating reliable cortisol detection across the dilution range. Dose–response analysis provided coefficients of determination (*R* ^2^) ranging from 0.972 to 0.998. The assay showed an analytical accuracy of 94.51 ± 4.35%. Intra‐assay CVs, calculated from repeated measurements within a single assay run (*N* = 8), were < 10%, whereas interassay CVs, calculated across different assay runs (*N* = 4), were < 15%, indicating acceptable assay precision. A summary of the assay validation parameters is presented in Table 2. Biological validation showed that cortisol levels were significantly higher during medical treatment (40.33 ± 12.15 ng/mL) than after recovery (22.12 ± 13.79 ng/mL), with the difference being statistically significant (*p* < 0.01; Figure 2(b)).

FIGURE 2(a) Parallelism test showing serial dilutions of Sumatran elephant serum (white circles and black triangles) compared with cortisol standards (black circles). (b) Cortisol levels during medical treatment and after recovery.

**Figure:**
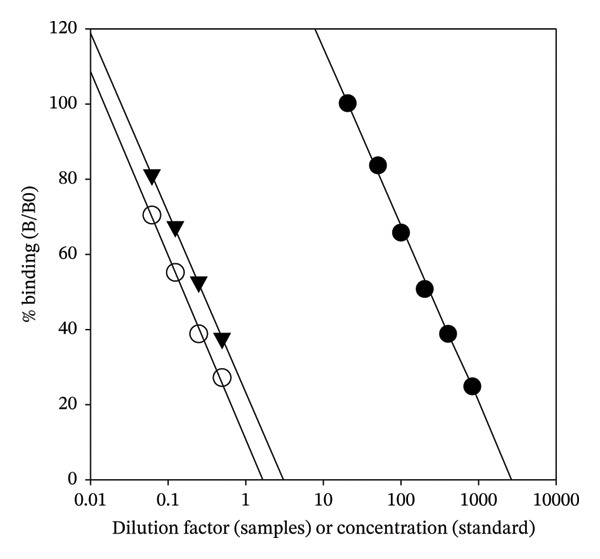
(a)

**Figure:**
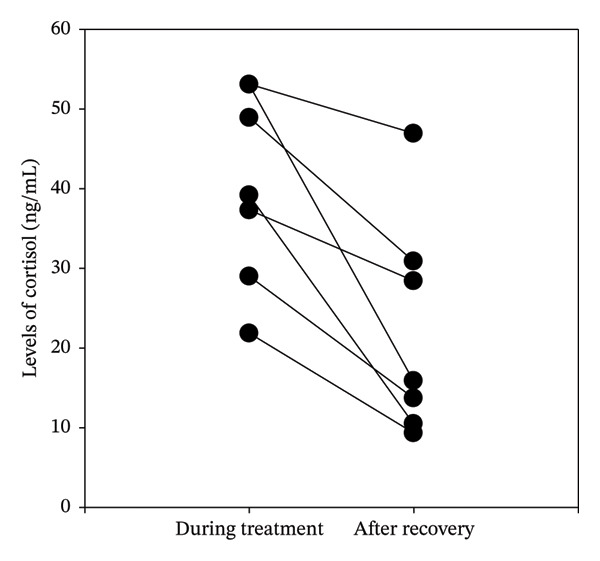
(b)

**TABLE 2:** Results of the analytical validation of the cortisol ELISA kit for cortisol measurement in the Sumatran elephant.

Measured parameters	Results
Parallelism	
‐Standard with Sample 1	Parallel, *z* = 0.875, *p* = 0.382
‐Standard with Sample 2	Parallel, *z* = 1.114, *p* = 0.266
Dose–response curve	
‐Standard	*Y* = −23.242 Ln (*x*) + 172.033 *R* ^2^ = 0.972, *p* = 0.001
‐Sample 1	*Y* = −21.304 Ln (*x*) + 10.554 *R* ^2^ = 0.996, *p* = 0.002
‐Sample 2	*Y* = −21.012 Ln (*x*) + 23.065 *R* ^2^ = 0.998, *p* = 0.001
Accuracy ± SD (%)	94.51 ± 4.35
Coefficient of variation (CV) of intra‐assay (%)	
‐Low‐quality control (*N* = 8)	9.52
‐High‐quality control (*N* = 8)	5.14
Coefficient of variation (CV) of interassay (%)	
‐Low‐quality control (*N* = 4)	10.13
‐High‐quality control (*N* = 4)	9.25

### 3.2. Associations of BCS and Location With Cortisol Levels

Results of the LMM showed a significant association between BCS and location with cortisol levels in Sumatran elephants at CRUs in Aceh. BCS was significantly associated with cortisol levels (F_3,74.30_ = 19.83, *p* < 0.001). Elephants with BCS 7 showed significantly lower cortisol levels than those observed in elephants with BCS 4, 5, and 6 (*p* < 0.001). Moreover, no significant differences were detected among BCS 4, 5, and 6 (Table 3).

**TABLE 3:** Estimated marginal means of cortisol levels across body condition score categories in Sumatran elephants.

Body condition score	Estimated mean cortisol (ng/mL)	SE	95% CI lower	95% CI upper
4	37.25^a^	7.51	22.28	52.22
5	49.63^a^	5.94	37.79	61.47
6	47.69^a^	3.39	40.93	54.46
7	18.28^b^	2.94	12.41	24.15

^a,b^Different superscripts above the estimated mean cortisol values indicate significant differences between groups (*p* < 0.05).

**FIGURE 3:**
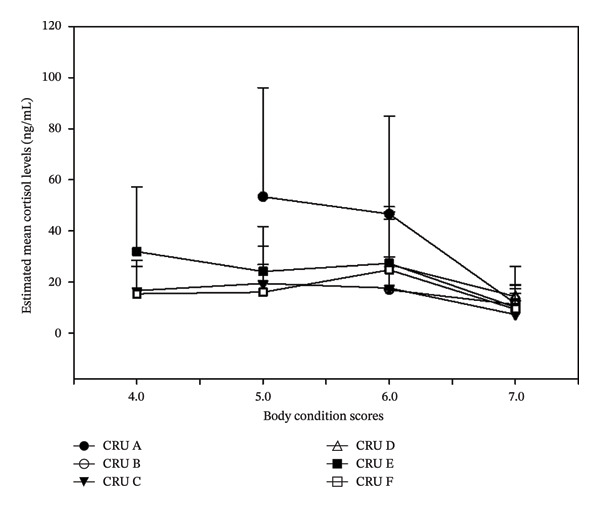
Interaction between body condition score (BCS) and location on estimated marginal mean cortisol levels (mean ± SE) in Sumatran elephants.

Location was also significantly associated with cortisol levels (F_5,62.70_ = 7.16, *p* < 0.001). Results showed that elephants at CRU A exhibited significantly higher cortisol levels compared to those observed at CRU B, C, D, E, and F (*p* < 0.05), while no significant differences were detected among CRU B, C, D, E, and F following Bonferroni correction (Table 4). A significant interaction between BCS and location was detected (F_10, 73.12_ = 3.05, *p* = 0.003), indicating that cortisol levels across BCS categories varied among locations (Figure 3). Variance component estimates indicated substantial residual variability (*σ* ^2^ = 185.58) and moderate between‐individual variability, supporting the appropriateness of the mixed‐effects modeling framework.

**TABLE 4:** Estimated marginal means of cortisol levels across conservation rescue units.

Conservation rescue units	Estimated mean cortisol (ng/mL)	SE	95% CI lower	95% CI upper
A	66.68^a^	6.28	54.17	79.19
B	24.31^b^	6.95	10.41	38.21
C	26.24^b^	5.96	14.35	38.13
D	36.80^b^	6.23	24.33	49.26
E	41.44^b^	3.75	33.87	49.01
F	28.28^b^	4.73	18.81	37.76

^a,b^Different superscripts above the estimated mean cortisol values indicate significant differences between groups (*p* < 0.05).

### 3.3. Associations of Ambient Temperature, Humidity, and THI With Cortisol Levels

Results of the LMM showed that ambient temperature (F_31,18.76_ = 3.22, *p* = 0.005), humidity (F_30,46.13_ = 4.082, *p* < 0.001), and THI (F_39,34.36_ = 6.71, *p* < 0.001) were significantly associated with cortisol levels. Across the observed temperature range (30.1°C–34.2°C), cortisol level generally increased with higher ambient temperatures (Figure 4(a)). Moreover, cortisol levels tended to be higher at relatively low humidity, although elevated cortisol was also observed at high humidity (Figure 4(b)). Similarly, cortisol levels tended to increase with higher THI, with the highest levels observed at elevated THI values, although some variability was present at intermediate levels (Figure 4(c)).

FIGURE 4Association between ambient temperature (a), humidity (b), and temperature–humidity index (c) with estimated marginal mean cortisol levels (mean ± SE) in Sumatran elephants.

**Figure:**
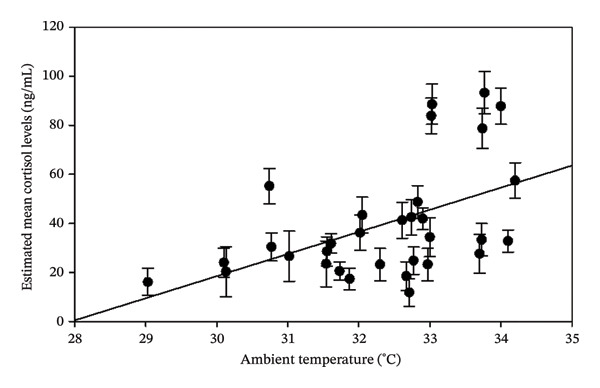
(a)

**Figure:**
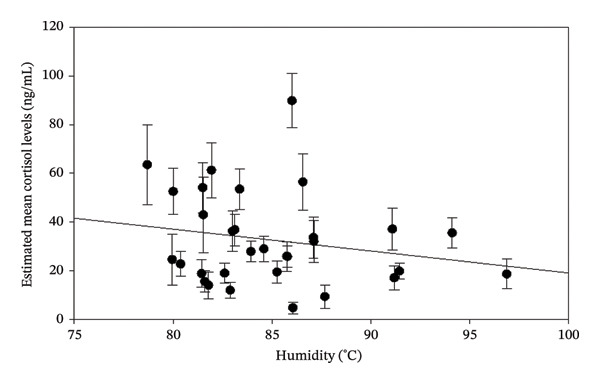
(b)

**Figure:**
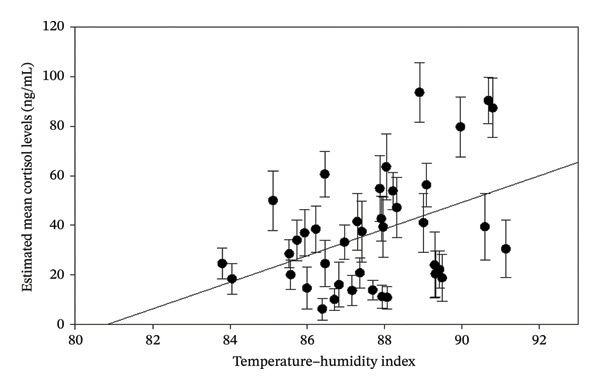
(c)

## 4. Discussion

This study provides a validation of a cortisol ELISA for use in Sumatran elephants under managed care conditions in CRUs and identifies associations between physiological stress, BCS, location, and ambient temperature. Analytical validation confirmed assay parallelism, accuracy, and precision, indicating reliable quantification of cortisol across a wide dilution range. Biological validation further supported assay sensitivity, as cortisol concentrations were significantly elevated during medical treatment compared with postrecovery conditions. Linear mixed‐effects modeling revealed that cortisol levels were significantly influenced by BCS, location, and ambient temperature, with notable interactions between BCS and location, highlighting the multifactorial nature of stress regulation in managed elephant populations.

The observed association between BCS and cortisol levels suggests that body condition may be related to variation in physiological stress in Sumatran elephants. Individuals with BCS 7 (scale 1–9) tended to exhibit lower cortisol levels, which may reflect a more favorable energetic and physiological state. Adequate body reserves may support metabolic homeostasis and contribute to reduced activation of the HPA axis through improved glucocorticoid feedback regulation [21]. In contrast, lower BCS may be associated with energetic deficits that could increase HPA axis activity, potentially through elevated corticotropin‐releasing hormone (CRH) and adrenocorticotropic hormone (ACTH) signaling, resulting in higher cortisol secretion [12, 22].

Evidence from both elephants and other animal species suggests that both low and excessively high body condition may influence stress physiology. In free‐ranging Asian elephants, glucocorticoid levels have been reported to be highest in individuals with very low BCS (e.g., BCS 1, scale 1–5) and to decline significantly toward moderate BCS levels (e.g., BCS 3, scale 1–5), after which values remain comparable for BCS 4 and 5 (scale 1–5) [23]. Similarly, studies in other species, such as goats, have shown that animals with moderate BCS (BCS 3, scale 1–4) exhibit lower cortisol levels compared to those with lower or higher body condition [24]. Those previous results are consistent with the present findings, where elephants with a moderate BCS (e.g., BCS 7, scale 1–9) showed lower cortisol levels compared to individuals with lower BCS. However, cortisol represents only one component of the stress response, and its relationship with BCS is likely influenced by additional environmental, physiological, and management‐related factors, as reported in previous studies in elephants and other species [23–26].

Location‐specific differences in cortisol levels indicate that variation among study sites may contribute to differences in physiological stress responses in Sumatran elephants. Elephants at CRU A exhibited higher cortisol levels compared to other locations, suggesting potential site‐related influences. As described in the Methods, the CRUs differed in habitat type and elevation, ranging from lowland forest and plantation‐associated landscapes to more forested and seminatural environments. These environmental differences may contribute to variation in resource availability and local conditions experienced by elephants. However, it is important to note that specific environmental and management variables (e.g., forage composition, intensity of human activity, or husbandry practices) were not directly quantified in this study. Therefore, differences in cortisol levels among locations should be interpreted cautiously and not attributed to a single factor.

Previous studies have shown that glucocorticoid levels in elephants are associated with environmental and anthropogenic factors. Higher cortisol levels have been reported in populations exposed to increased human disturbance and habitat modification, including plantation expansion (e.g., tea plantations) [26]. Similarly, glucocorticoid levels have been found to increase in human‐modified landscapes with greater anthropogenic activity and lower vegetation productivity [27–30]. These findings suggest the potential role of anthropogenic pressures in influencing stress physiology in elephants, although such factors were not directly measured in the present study.

The significant interaction between BCS and location suggests that the relationship between body condition and cortisol varies across sites. Elephants with lower BCS tended to exhibit higher cortisol levels at certain locations, whereas individuals with moderate BCS levels (BCS 7, scale 1–9) showed relatively lower cortisol concentrations across sites. This pattern may indicate that body condition is associated with variation in physiological stress, although this relationship appears to depend on site‐specific and animal conditions [31]. Previous studies have shown that glucocorticoid levels vary significantly among camps, likely reflecting differences in management practices such as feeding, activity levels, and tourism intensity [32]. In addition, elephants rely on behavioral and physiological adaptations, such as shade use and wetting, to cope with environmental conditions, although the effectiveness of these responses depends on the availability of resources such as water, forage, and shade in their habitat [33].

Given the observed interaction between BCS and location, site‐specific environmental factors may contribute to variability in cortisol responses across CRUs. In this context, ambient temperature, humidity, and THI were identified as significant predictors of cortisol levels. Ambient temperatures may act as an environmental factor influencing physiological responses in elephants and other animals, particularly under conditions of increased thermoregulatory demands [10–12, 20, 34]. Humidity may further modulate thermal stress by affecting evaporative heat loss efficiency, thereby influencing the animal’s ability to dissipate body heat [11, 35]. Under high humidity conditions, reduced evaporative cooling can exacerbate heat load, while low humidity may also impose physiological challenges depending on the environmental context. This may explain the observed variability in cortisol levels across humidity gradients. The combined effect of temperature and humidity, as reflected by THI, provides a more integrated measure of thermal load [20, 35]. The significant association between THI and cortisol levels in the present study suggests that cumulative heat stress, rather than single environmental factors alone, plays a critical role in shaping endocrine stress responses. This is consistent with previous studies indicating that large mammals, including elephants, rely on complex anatomical, physiological, and behavioral mechanisms to maintain thermal balance under varying climatic conditions [11, 20].

Previous studies have shown that elephants exhibit behavioral and thermoregulatory adjustments in response to heat stress, including increased use of shade and water‐related behaviors to regulate body temperature [20, 34]. In addition, experimental findings from other biological systems further suggest that heat exposure can trigger cellular stress responses, such as oxidative stress and metabolic disruption [36]. Variability in glucocorticoid levels has also been reported to be influenced by multiple environmental and management‐related factors in Asian elephants, highlighting the multifactorial nature of stress responses [4, 6, 37, 38]. Consistent with these findings, the present study identified a positive association between ambient temperature and cortisol levels, suggesting that thermal conditions may be one of several factors contributing to variation in stress‐related endocrine responses. However, this relationship should be interpreted cautiously, as cortisol represents only one component of the stress response and may also be influenced by additional environmental and physiological factors.

Previous studies have reported that glucocorticoid levels in elephants vary in response to a range of physiological, environmental, and management‐related factors. For example, glucocorticoid activity has been shown to fluctuate with physiological changes and social life events in Asian elephants [3], and to exhibit substantial plasticity across individuals and contexts in both African and Asian elephants [6]. Body condition and environmental conditions, including seasonal and climatic variation, have also been associated with changes in glucocorticoid levels [12, 23]. In addition, management‐related factors such as tourist activities and husbandry practices may influence adrenal and metabolic responses in captive elephants [31]. These findings suggest that glucocorticoid responses in elephants are multifactorial and context‐dependent. In the present study, associations between cortisol levels, body condition, and ambient temperature were observed, consistent with previously reported factors. These interpretations are supported by an analytical approach that accounts for repeated measurements and variation among individuals and locations, consistent with approaches used in longitudinal physiological studies [39, 40].

From a theoretical perspective, the present findings may be interpreted within the allostatic load framework, which describes how exposure to environmental and physiological challenges can influence stress‐regulatory systems [41, 42]. In this context, body condition may reflect aspects of energetic balance and health status, while environmental factors such as ambient temperature and habitat condition may act as external influences on adrenal activity [43]. However, detailed information on management practices and fine‐scale habitat characteristics (e.g., forage availability and human–elephant conflict) was not explicitly quantified in this study, and their potential contributions should be interpreted with caution [6]. Overall, the present results contribute to a growing body of work examining factors associated with stress physiology in elephants and may inform future studies that incorporate longitudinal endocrine data alongside more detailed ecological and management variables.

## 5. Conclusion

In conclusion, this study validated the use of a cortisol ELISA for Sumatran elephants, demonstrating acceptable analytical and biological performance for e cortisol measurement. Application of the assay showed that BCS, ambient temperature, relative humidity, and the THI were associated with variations in cortisol levels. The results provide useful baseline information for elephant welfare monitoring and may support the development of management strategies that consider body condition and environmental factors, including composite thermal indices such as THI, as important components of conservation and husbandry practices, particularly at CRUs in Aceh, Indonesia.

## Author Contributions

Each author contributes equally to this study. Conceptualization: Gholib Gholib and Arman Sayuti; methodology: Gholib Gholib, Riyan Ferdian, and Arman Sayuti; data curation: Riyan Ferdian, Ridwan Ridwan, and Arman Sayuti; data analysis: Gholib Gholib and Taufiq Purna Nugraha; resources: Gholib Gholib and Cristopher R. Stremme; writing–original draft: Gholib Gholib and Arman Sayuti; writing–review and editing: Gholib Gholib, Yudha Fahrimal, Amalia Sutriana, and Gono Semiadi.

## Funding

This research was supported by the RIIM LPDP Grant (Research and Innovation for Advanced Indonesia, funded by the Indonesia Endowment Fund for Education/LPDP) and the National Research and Innovation Agency (BRIN), Indonesia (Grant No. 79/IV/KS/05/2023 and 22/UN11.2.1/HK.07.00/2023).

## Disclosure

All authors have read and agreed to the published version of the manuscript.

## Conflicts of Interest

The authors declare no conflicts of interest.

## Data Availability

The data supporting the findings of this study are available from the corresponding author.
